# Nondual Awareness, Body Boundary Perception, and Creative Attitudes in Japanese University Students Through a Brief Paired Martial Arts Exercise: A Randomized Controlled Trial

**DOI:** 10.3390/bs15121638

**Published:** 2025-11-28

**Authors:** Arisa Yokosu, Takahiko Maruyama, Hiromitsu Miyata

**Affiliations:** 1 Graduate School of Letters, Arts and Sciences, Waseda University, Tokyo 162-8644, Japan; 2 School of Culture, Media and Society, Waseda University, Tokyo 162-8644, Japan; taka.maruyama@aoni.waseda.jp; 3 Faculty of Letters, Arts and Sciences, Waseda University, Tokyo 162-8644, Japan; miyata@waseda.jp

**Keywords:** martial arts, wakame exercise, nondual awareness, self–other boundary, creative attitude, Eastern mind-body practice

## Abstract

Mind–body practices such as meditation, yoga, and martial arts have been suggested to enhance flexible self-experience and psychological well-being. However, few studies have examined short-term effects of contemplative bodywork rooted in traditional martial arts on perception of self–other boundaries and relevant psychological states. The present study employed a three-arm randomized controlled design to examine psychological effects of the Wakame Exercise, a paired body–mind practice derived from a school of martial arts in Japan. Seventy undergraduates were randomly assigned to a 45 min session of wakame exercise (*N* = 25; 20 females, 5 males; *M* age = 19.4, *SD* = 0.64), a control practice, Push-Hand Sumo (*N* = 25; 15 females, 10 males; *M* age = 19.5, *SD* = 1.20), or rest (*N* = 20; 17 females, 3 males; *M* age = 19.4, *SD* = 1.09). Outcomes included nondual awareness, perceived body boundaries, creative attitudes, decentering, and positive/negative affect. The wakame exercise group showed significant increase in nondual awareness (*p* < 0.001, Cohen’s *d* = −1.74) and decrease in the salience of perceived body boundaries (*p* < 0.001, Cohen’s *d* = 1.25) following practice, which were more apparent than controls. Within the wakame exercise group, nondual awareness was significantly positively correlated with creative attitudes, and higher creative attitudes were associated with greater decentering following practice. These findings suggest that brief paired practices rooted in Eastern martial arts can promote nondual awareness and temporary boundary dissolution, potentially enhancing creativity (Approved by the Ethics Review Committee on Research with Human Subjects of Waseda University, No. 2022–397).

## 1. Introduction

Traditional Eastern body–mind practices, including martial arts, zazen meditation, qigong, Tai Chi, and yoga, have gained academic attention for their potential to transform both mental and physical status. These practices are suggested to not merely involve physical movements but also incorporate meditative elements and self-cultivation, often framed as a “Way” (dō), in which mind and body are developed in an integrated manner (Yuasa, 1990). A central feature of Eastern body–mind practices is the cultivation of a state in which the body is experienced as being continuous with others and the surrounding world, i.e., a state referred to as a “pre–subject–object” mode of bodily awareness (Yuasa, 1990). For example, in Aikido, a form of Japanese traditional martial arts, practitioners seek to harmonize with an opponent’s movements rather than resist them forcefully, to transcend the dichotomy between the self and the other (Kudo & Shishida, 2010). Following Ciaccioni et al. (2023), Moore et al. (2020), and Maliszewski (1992), martial arts are considered to be understood as both systems of combat techniques and embodied methods of self-cultivation, i.e., practices aimed at regulating and harmonizing physical and mental energy. Cynarski (2016) proposed the General Theory of Fighting Arts (GTFA) as a theoretical framework to understand martial arts not merely as competitive sports but rather as a comprehensive academic subject encompassing cultural, philosophical, and ethical dimensions. The term “martial arts” is inherently dual in that it involves not only fighting skills but also cultivation of mind–body health and philosophical development (Maliszewski, 1992; Pawlucki, 2023). In such sense, martial arts can be regarded simultaneously as physical disciplines and as embodied approaches to personal growth and well-being.

Empirical studies have suggested that both Tai Chi and qigong can enhance parasympathetic activity, reduce heart rate and blood pressure, improve respiratory functions, and potentially reduce oxidative stress (Goon et al., 2009; Wang et al., 2013). Tai Chi practice has also been found to alleviate depression and anxiety as well as to increase psychological well-being, self-esteem, and life satisfaction (Priddy et al., 2018; Skrzeta et al., 2021; Wang et al., 2013). Qu et al. (2024) reported that mindfulness-enhanced Tai Chi programs showed greater improvements in mindful awareness and stress reduction compared with traditional forms of Tai Chi.

In Japan, martial arts have historically been practiced both as techniques of combat and as disciplines for holistic self-development, and training has been associated with emotion regulation, self-esteem, and positive personality traits (Burke et al., 2007; Moore et al., 2020). For example, practice of aikido emphasizes breathing, balance, centering, and connection with a partner (Kudo & Shishida, 2010), which are deemed to cultivate non-judgmental awareness comparable to mindfulness (Lothes et al., 2013). According to Miyata et al. (2020), continued practitioners of a school of martial arts based on Japan’s traditions, including swordsmanship, karate, and bojutsu, self-reported significantly higher dispositional mindfulness and subjective well-being than non-practitioners. Among the practitioners, length of practice was significantly associated with higher scores on these scales. These findings suggest that engaging in traditional Japanese martial arts can have beneficial impacts on psychological health (see also Miyata et al., 2021).

A recent systematic review suggests that both martial arts and combat sports are associated with improved cognitive control, response inhibition, and emotional regulation, although findings vary depending on the style and duration of these practices (Ciaccioni et al., 2023; Moore et al., 2020). Previous research on martial arts has primarily focused on long-term practice (e.g., several weeks to months) or competitive practice (Finkenberg, 1990; Trulson, 1986). That is, little is known about the short-term psychological effects of paired, martial-arts-based interventions, particularly with respect to their potential to induce transient experience such as nondual awareness and/or temporary dissolution of self–other boundaries. Therefore, in this frontier, there is a need to accumulate evidence on short-term psychological impacts of paired exercises in non-competitive and/or fighting contexts.

From a theoretical perspective, Eastern philosophical traditions have long conceptualized the self as being dynamic and interdependent, in contrast to Western notions of an autonomous, bounded ego. Buddhist perspectives, for instance, typically deny the existence of a permanent self and describe awareness beyond the subject–object dichotomy (Gallagher et al., 2024; Kelly, 2008; Zoran, 2019). In the context of psychological studies, such state of consciousness is referred to as nondual awareness (NDA). NDA involves an experiential dissolution of self–other boundaries, often described in meditative contexts as expansion into space, merging with the environment, or a sense of formless awareness (Gyamtso, 1994; Hanley et al., 2018; Nguyen, 2019; Zoran, 2019).

In Western psychiatry, such fluidity of self–other boundaries is often considered to reflect psychopathological status (Federn, 1952) such as depersonalization (Nave et al., 2021). However, thinness of self–other boundaries has also been positively linked to heightened sensitivity and creativity (Hartmann, 1991). Psychoanalytic theorists have coined the term “creative regression” to denote a temporary loosening of ego boundaries that can enable intuitive insight, so long as self-regulatory capacities are well maintained (Kris, 1952; Schafer, 1954). In addition, previous studies suggest that boundary-dissolving experiences are not limited to advanced practitioners of meditation or martial arts (Nave et al., 2021). Even novices can undergo temporary shifts in self–other boundaries. According to the theory of creative regression (Schafer, 1954), when the loosening of self–other boundaries occurs while basic self-functions remain intact, fluidity of self–other boundaries can enhance psychological flexibility and creativity. Under such conditions, decentering, i.e., the metacognitive ability to observe one’s thoughts and emotions as transient mental events without over-identifying with them (Fresco et al., 2007), may allow individuals to maintain functional adaptation to reality while simultaneously relaxing rigid selfother boundaries, thereby facilitating creativity.

Within these contexts, the present research focuses on the “*Wakame Exercise*,” a basic paired practice derived from a contemporary school of martial arts based on Japanese traditions (Aoki, 1997). The wakame exercise intends to enhance soft and flexible movements of the body, aiming to facilitate mental unification of the self and the opponent. Such exercise may thus allow practitioners to experience nondual awareness and thinning of perceived body boundaries, in a way that can enhance creative attitudes and positive affectivity. The wakame exercise is safely applicable to the populations of non-practitioners of martial arts such as university students with good instructions, and may thus provide a notable example of how short-term paired practice based on Eastern bodywork may induce such psychological effects.

## 2. Purpose of the Present Study

Building upon these theoretical and empirical insights, the present study involving a sample of Japanese university students aimed to examine whether and how a short-term practice of the wakame exercise can induce changes in psychological states including nondual awareness, perception of self–other boundaries, creative attitudes, decentering, and positive/negative affectivity. Based on the practical implications of the wakame exercise to dissolve self–other boundaries, we formulated the following hypotheses: (1) practice of the wakame exercise would induce changes in psychological states including enhanced state nondual awareness, reduced salience of perceived body boundaries, and enhanced creative attitudes and decentering, as well as increased positive affect and decreased negative affect. (2) Among individuals who practiced the wakame exercise, associations between psychological state variables would be observed immediately after completing the practice, such that nondual awareness would be positively associated with creative attitudes and positive affect, and creative attitudes would be positively associated with decentering and positive affect. (3) Among individuals who practiced the wakame exercise, general boundary perception at the trait level may be associated with psychological states when completing the practice; for example, individuals with a thinner general sense of boundary may report higher nondual awareness, lower salience of body boundary perception, and/or higher creative attitudes.

## 3. Materials and Methods

### 3.1. Participants

A total of 70 undergraduate students (52 females, 18 males; age range = 18–23 years, *M* = 19.5 years, *SD* = 1.00) who belonged to a private university located in Tokyo, Japan, participated in the study. There were no participants who did not report their sex. All participants were enrolled in one of the three undergraduate seminars on psychology held by the third author and participated as part of the course activities. For the randomized controlled trial (RCT), participants were randomly assigned to one of the three groups by using a stratified randomization procedure to ensure an approximately balanced distribution of sex across groups: (1) the wakame exercise group as an experimental group (*N* = 25; 20 females, 5 males; age range = 19–21 years, *M* = 19.4 years, *SD* = 0.64), (2) the “*Push-Hand Sumo*” group an active control (*N* = 25; 15 females, 10 males; age range = 19–23 years, *M* = 19.5 years, *SD* = 1.20), and (3) the rest group as a passive control (*N* = 20; 17 females, 3 males; age range = 18–23 years, *M* = 19.4 years, *SD* = 1.09). A chi-square test for equivalence revealed that there was no significant difference in gender distribution across the groups (*χ*^2^ [4] = 6.000, *p* = 0.199). There was no duplication of participants in the three data collection sessions. Participants were not compensated for cooperation in the study.

### 3.2. Psychological Scales

#### 3.2.1. Japanese Boundary Questionnaire (JBQ; Kodama, 2013)

The JBQ, adapted from Hartmann’s (1991) Boundary Questionnaire, was developed by Kodama (2013) to be culturally appropriate for Japanese populations. The questionnaire assesses various aspects of psychological boundaries experienced in daily life, such as the permeability of external stimuli, relationship between dreams and wakefulness, body image boundaries, interpersonal distance, environmental preferences, etc. The JBQ evaluates the thinness or thickness of boundaries as a personality trait. The JBQ consists of 52 items each rated on a 5-point Likert scale from 0 (does not apply at all) to 4 (applies very much), and a total score is calculated. Higher scores indicate thinner, more permeable psychological boundaries in daily life, whereas lower scores indicate thicker, less permeable boundaries. Internal consistency score (Cronbach’s α) in the present sample was 0.79.

#### 3.2.2. Nondual Awareness Dimensional Assessment-State (NADA-S; Hanley et al., 2018)

The NADA-S is a state scale for evaluating “Nondual Awareness (NDA).” NDA denotes views of the world and the self as fundamentally one, without separating the subject from the object, a concept that has been explored in both Eastern and Western mystical traditions. Because a Japanese version of the scale was not available at the time of the study, the first author independently translated the items into Japanese for administration. The items included: “*I experienced the boundaries of myself dissolving*,” “*I experienced my mind expanding into space*,” “*I experienced all things seeming to unify into a single whole*,” “*I experienced all sense of self and identity dissolve away*,” and “*I felt surrounded and filled with a blissful warmth or energy*.” These items assess the extent to which the boundaries between self and other are being blurred and integrated. Responses to each item were rated on a 10-point Likert scale from 1 (not at all) to 10 (very much), with higher scores indicating stronger experiences of nondual awareness and a greater tendency to perceive self and other as unified, and lower scores indicating weaker experiences of nondual awareness. The original NADA-S comprises two factors (i.e., self-transcendence, bliss); however, this was deemed theoretically consistent with the unidimensional construct of nondual awareness, which the present study conceptualizes as a coherent experiential state rather than separable dimensions. In the present study, a confirmatory factor analysis was conducted to examine the factor structure of the present sample. One-factor model was adopted, and a total score for all five items was calculated. Cronbach’s α was 0.74 and 0.90 for the pre- and post-session measurement, respectively.

#### 3.2.3. Perceived Body Boundary Scale (Based on Dambrun, 2016)

**Figure 1:**
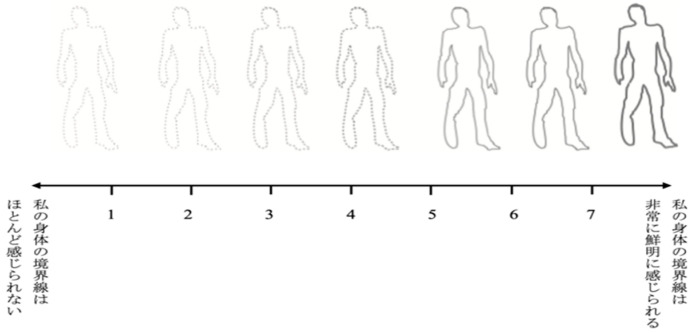
Illustrations used for the Perceived Body Boundary Scale. The Japanese sentences on the left and right ends read “*My body boundaries are almost imperceptible*.” and “*My body boundary are extremely salient*,” respectively.

To measure the salience of bodily boundaries, a single-item, visualized scale for perceived body boundary was used (Figure 1). Ataria et al. (2015) proposed that the sense of bodily boundaries is a dynamic experience that exists on a spectrum, ranging from highly salient bodily boundaries to scarcely perceptible ones. The Perceived Body Boundary Scale is based on such views and emphasizes that bodily boundaries are conceived more flexibly rather than being limited to the physical contour of the body. In the present study, to facilitate participants’ understanding of the scale, we provided the following instruction in Japanese: “A clearly perceived bodily boundary refers to the sensation that one’s body is surrounded by a distinct contour, clearly separated from others’ bodies, and demarcated from the external environment. In contrast, an unperceived bodily boundary refers to the sensation that one’s body is strongly connected to the surrounding environment, without a distinct separation from it, as if integrated with the environment. Which of the following statements 1 through 7 best describes you right now? If you feel your body’s boundaries very distinctly, select 7. If you feel your body’s boundaries barely at all, select 1.” Responses were rated on a 7-point Likert scale from 1 (“*I can hardly feel the boundaries of my body*”) to 7 (“*I can clearly feel the boundaries of my body*”), with higher scores indicating that participants perceived their bodily boundaries as more distinct and salient, and lower scores indicating that participants experienced their bodily boundaries as thinner or less clearly defined.

#### 3.2.4. Creative Attitude Questionnaire

Creative attitudes associated with meditative experiences were assessed using a brief questionnaire originally developed by the authors based on Onda (1995). The question items were designed to assess creativity-related psychological states that are characteristic of meditative states as denoted by Onda (1995), such as tolerance for ambiguity, openness to change, and prioritization of intuition over conventional values. Based on these concepts, the following 4 items were used: “*I do not stick to the existing way of doing things, and I am not afraid to change my behavior according to the situation*,” “*I am eager to learn more about new knowledge and the unknown*,” “*I can accept uncertainty and contradiction even if confronted with them*,” and “*I prefer to value how I intuitively feel rather than existing values or general evaluations*.” Responses to each item were rated on a 10-point Likert scale from 1 (I do not agree at all) to 10 (I agree very much), with higher scores indicating greater creativity associated with meditative states. A confirmatory factor analysis was conducted to examine the factor structure, and a total score across all four items was calculated for each participant. Cronbach’s α was 0.82 and 0.75 for the pre- and post-session measurement, respectively.

#### 3.2.5. Japanese Version of the Experiences Questionnaire (J-EQ; Kurihara et al., 2010)

The J-EQ was used to assess decentering during the practical session of the wakame exercise. The J-EQ consists of two subscales: *Decentering* (10 items) and *Rumination* (5 items). Items that fall into the decentering subscale assess the capacity to accept experiences from an objective perspective, such as “*I can detach myself from my thoughts and feelings.*” and “*I have a sense of being fully aware of what is happening around me and inside myself.*” Responses to each item are rated on a 5-point Likert scale ranging from 1 (not applicable) to 5 (applicable). All items of the original scale, including the decentering and rumination subscale, were administered. For the present study, scores from the rumination subscale were excluded from analyses because they were not relevant to the study’s aims, and only the total scores of the decentering subscale were used. Higher scores on the decentering subscale indicate a greater tendency to distance oneself from one’s experiences and emotions and to observe them, whereas lower scores indicate a greater likelihood of being absorbed in experiences and emotions, with a reduced tendency for objective observation. Cronbach’s α was 0.81 and 0.87 for the pre- and post-session measurement, respectively.

#### 3.2.6. Positive and Negative Affect Schedule (PANAS; Sato & Yasuda, 2001)

The PANAS (Watson et al., 1988) is a brief, widely used mood rating scale. The Japanese version of the PANAS (Sato & Yasuda, 2001) was used. The PANAS is a 16-item scale comprising two subscales: 8 items for Positive Affect (PA; e.g., “Active,” “*Excited*,” etc.) and 8 items for Negative Affect (NA; e.g., “Afraid,” “*Irritable*,” etc.). In the present study, the PANAS was used as a state scale and participants were instructed to report their current mood states. Participants rated each item on a 6-point Likert scale ranging from 1 (“*not at all*”) to 6 (“*very much*”). In the present sample, Cronbach’s α was 0.86 for the PA and 0.88 for the NA, respectively.

### 3.3. General Procedure

Data collection was conducted during three separate sessions on different dates (12 May 2023, 19 October 2023, and 14 June 2024). Each session for data collection corresponded to one class of the seminar course for undergraduate students. At the beginning of each class, the researchers (first and third authors) distributed the paper-based, pre-session questionnaire sheets to the participants and orally explained the content of the study, including the overview of the study as well as information on the protection of personal information. Participants were also clarified that both participation in the assigned activities and cooperation in the questionnaire survey were voluntary and that they did not affect the grading of the course. In addition to the demographic information, the pre-session survey involved a question of whether the participants engaged in daily practice of Eastern tradition-based mind–body practices such as mindfulness, meditation, martial arts, yoga, etc. The survey also included the JBQ and the state questionnaires. After the researchers (first and third authors) explained the content of the study, participants completed the pre-session survey. Participants were then informed of their group assignment and were directed to separate locations within the same campus according to their assigned group.

Randomization and group allocation were conducted by the first and third authors, both of whom analyzed the data after the sessions. To minimize potential bias regarding group assignment, randomization of participants within each class was conducted by sequentially allocating three different symbols (i.e., A, B, and C) in order from the top of the attendance list. Then, the allocation was minimally adjusted as necessary so that the number of male and female participants was balanced across the groups, while ensuring that same-gender pairs could be formed for both the wakame exercise and push-hand sumo groups. Specific content of each exercise was disclosed to the participants only after they had arrived at the assigned room.

Participants in the wakame exercise group were guided by the second author, and those in the push-hand sumo group were guided by the first author. Participants in the rest group were instructed to move to the library located within the university campus, which all participants were familiar with. All participants were unaware of the hypotheses regarding group assignment (i.e., single-blinded), whereas the researchers knew how group assignment was conducted. Each practical session for each group lasted for 45 min, and the total duration of the session, including instructions and completion of the pre- and post-session questionnaire surveys, was approximately 100 min. Immediately after the practical session had ended, all participants were sent back to the original room and were instructed to complete the post-session questionnaire survey. The post-session survey included psychological state questionnaires identical to those administered before the practical session.

### 3.4. Intervention for Each Group

#### 3.4.1. Wakame Exercise Group

**Figure 2:**
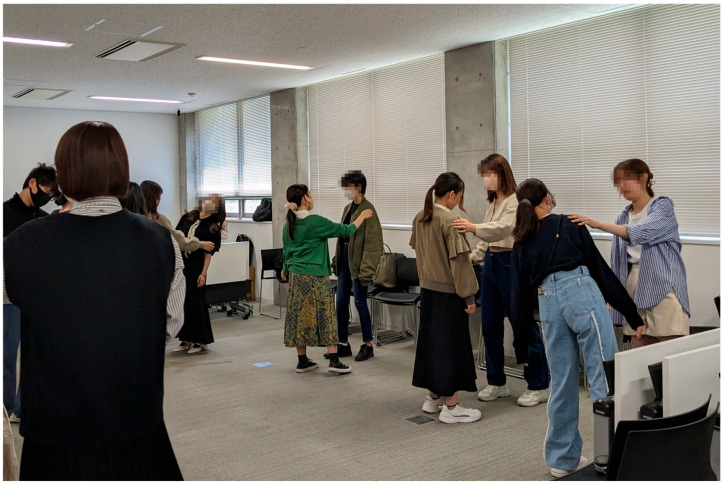
A photo during a practical session of the wakame exercise. The photo was taken by the first author in May 2023.

The “*Wakame (Seaweed) Exercise*,” called “*Wakame Taiso*” in Japanese, is one of the basic methods of practice in *Tenshintaido*, i.e., a school of martial arts founded by Hiroyuki Aoki based on Japanese traditions. The wakame exercise is also called a meditative *kumite* (sparring practice), because of its contemplative nature to emphasize acceptance of the present moment and to promote a natural state of being (Aoki, 1997). According to Aoki (1997), as a basic practice focusing on the cultivation of soft and flexible bodily movements, the wakame exercise can facilitate mental unification and allow practitioners to experience the essential aspects of martial arts. The exercise can be safely practiced by individuals regardless of prior experience with Eastern mind–body practices. In the wakame exercise, practitioners are assigned to one of two roles: the “*wave-player*” or the “*wakame-player*.” The wave-player gently applies pressure to the wakame-player’s shoulders and back, while the wakame-player moves their body softly, surrendering to the sensations and movements generated by the wave-player (Figure 2). As the practitioners repeatedly engage in such interactions while switching the roles, the distinction between self and other is assumed to fade gradually, evoking a meditative sense of fusion. In the present study, pairs were formed within the same sex to avoid direct physical contact between opposite sexes, in consideration of cultural norms and ethical appropriateness within Japanese university settings. The instruction of the wakame exercise was conducted by the second author, who was an experienced martial arts practitioner belonging to the Kenbu Tenshin-Ryu school, which primarily focused on swordsmanship. The instructor served as a certified Shihan at the Kenbu Tenshin-Ryu headquarters until 2023, and has held the rank of Shihan (4th Dan) since 2024.

For the practical session, the instructor first gave a brief explanation of traditional martial arts including background of the establishment of the wakame exercise. Participants then engaged in simple warm-up activities to relax their bodies (i.e., bending and stretching, body shaking, and body scanning) and then conducted the wakame exercise in pairs. After each pair of participants experienced the wave-player and wakame-player roles alternately, the pairs were rearranged, and the same exercise was repeated multiple times within the timeframe. Because the wakame exercise consists of simple body movements, involves low physical intensity, and emphasizes mild interpersonal attunement rather than technical expertise, the exercise can be safely performed even by individuals without prior experience in martial arts or other Eastern mind–body practices.

#### 3.4.2. Push-Hand Sumo Group

To control for the physical and motor components of the wakame exercise, the “*Push-Hand Sumo Group*” was introduced as an active control. The push-hand sumo, called “*Te-oshi Zumo*” in Japanese, is a paired physical activity in which participants face each other and use their hands to unbalance their opponent (Kurokawa, 2018). The push-hand sumo is apparently similar to the wakame exercise in that it is a low-intensity physical activity and involves a paired execution; however, the push-hand sumo focuses on a win–lose competition and does not include contemplative and/or Eastern philosophical components that are inherent in the wakame exercise. Prior to the push-hand sumo activity, participants were briefly explained about potential benefits of push-hand sumo for physical conditioning, particularly enhancement of core strength (Koba, 2021). Warm-up exercises during a practical session included trunk-focused breathing and balance exercises (e.g., standing on one leg with eyes closed). Participants then engaged in push-hand sumo in same-sex pairs and repeated the matches within the timeframe. As in the wakame exercise group, pairs were formed within the same sex to avoid cross-gender physical contact.

According to Wong et al. (2013), Tai Chi push-hand exercises, in which two participants push against each other’s hands, generate low impact forces and are deemed suitable for older adults. Similarly, Kurokawa (2018) described the push-hand sumo as a paired hand-pushing activity involving physical exertion comparable to Tai Chi push-hand exercises. Based on such literature, we assumed that the push-hand sumo is a light-intensity physical exercise comparable to the wakame exercise. That is, the push-hand sumo involves the paired hand-pushing movement components, parallel to the wakame exercise; however, it lacks contemplative and/or philosophical dimensions as are the essential parts of the wakame exercise.

#### 3.4.3. Rest Group

The Rest Group was introduced as another, passive control. Participants assigned to the rest group were instructed to spend their time quietly in the library as they normally would. During this period, they were not instructed to engage in any contemplative practices or exercises. Participants visited the library, stayed there during the same period as the other groups, and returned to the original classroom at the end of the session.

### 3.5. Data Analysis Methods

No participants withdrew participation or were excluded after randomization, and data from all 70 participants (*N* = 25, 25, and 20 for the wakame exercise, push-hand sumo, and rest groups, respectively) were included in the analysis. Because a priori power analysis was not conducted prior to data collection, post hoc power analyses by using G*Power (Version 3.1.9.6) were conducted both for the overall statistical power and for the achieved power for each total/subscale score from the psychological scales. There were 3 missing items across all the psychological scales and measurement points, which accounted for 0.05% of the data from psychological scales. Where there were missing items, the total/subscale scores for that participant were also treated as missing and were excluded from analysis.

For the NADA-S and the Creative Attitude Questionnaire, confirmatory factor analyses were conducted by using jamovi (Version 2.645) to examine the factor structure of each scale. For all scales on psychological states (i.e., all scales except for the JBQ), total scores were calculated separately for pre- and post-session assessments, and means and standard deviations (*SD*s) were subsequently computed for each group. To assess potential baseline differences for each psychological measure, data from 3 participants who reported regular practice of Eastern mind–body disciplines (e.g., meditation or martial arts) were compared with those from the remaining participants by using independent samples *t*-tests.

Next, to evaluate changes in psychological states from pre- to post-session assessments, a mixed-factor two-way analysis of variance (ANOVA) with group (3 levels: wakame exercise, push-hand sumo, rest) as a between-participants factor and measurement point (2 levels: pre- and post-session) as a within-participants factor was conducted for scores from each psychological state scale. These statistical analyses were run by using the download-free statistical software HAD Version 17 (Shimizu, 2016). Where an interaction between group and measurement point was statistically significant, multiple comparisons using the Holm method were further conducted to test pre–post differences within each group. Furthermore, among individuals from the wakame exercise group, Pearson’s correlation coefficients (Pearson’s *rs*) were calculated to examine associations between scores from the psychological state scales at the post-session assessment. In addition, correlation coefficients between total scores from the JBQ and scores from each psychological state scale were computed for both pre- and post-session assessments within each group.

## 4. Results

### 4.1. Post Hoc Power Analyses

Assuming a medium effect size (*f* = 0.25), the overall statistical power (1−*β*) with *N* = 70 was 0.432. The achieved statistical power (1−*β*) for each primary outcome calculated based on the observed effect sizes was 0.953 for the NADA-S, 0.777 for the Perceived Body Boundary Scale, 0.104 for the Creative Attitude Questionnaire, 0.053 for the decentering subscale from the J-EQ, 0.884 for the PA from the PANAS, and 0.395 for the NA from the PANAS. Thus, the sample provided a sufficient statistical power to detect differences for the NADA-S and the PA from the PANAS and a moderate power for the Perceived Body Boundary Scale, whereas the power was low for the remaining scales.

### 4.2. Confirmatory Factor Analyses

For the NADA-S, both the one-factor model (*χ*^2^ [5] = 10.679, *p* = 0.058; CFI = 0.946; TLI = 0.891; RMSEA = 0.127; AIC = 1432.045; BIC = 1465.772) and the two-factor model (*χ*^2^ [4] = 10.638, *p* = 0.031; CFI = 0.936; TLI = 0.840; RMSEA = 0.154; AIC = 1434.004; BIC = 1469.980) showed an acceptable fit. Because multiple goodness of-fit indices indicated a better fit of the one-factor model over the two-factor model, the one-factor model was adopted, consistently with the theoretical conceptualization of nondual awareness as a unidimensional experiential state.

For the Creative Attitude Questionnaire, the one-factor model showed an acceptable fit (*χ*^2^ [2] = 3.012, *p* = 0.222; CFI = 0.932; TLI = 0.796; RMSEA = 0.085; AIC = 1151.959; BIC = 1178.941). All factor loadings were positive and of reasonable magnitude (range: 0.868–1.338), indicating that the items coherently reflected a single underlying construct. Accordingly, the scale was treated as unidimensional, and a total score across the four items was calculated for subsequent analyses.

### 4.3. Participant Characteristics and Baseline Comparisons

Comparisons between regular practitioners of Eastern mind–body practices and non -practitioners revealed statistically significant differences for scores from the NADA-S (*t* (68) = −3.760, *p* < 0.001 Bonferroni-corrected) and the Perceived Body Boundary Scale (*t* (4) = −7.057, *p* = 0.002, Bonferroni-corrected). Thus, data from the 3 practitioners were excluded from analysis when calculating total scores from these scales. No statistically significant differences were found for other psychological measures (*t* = 0.029–0.899, all *p*s > 0.007, Bonferroni-corrected).

### 4.4. Pre- to Post-Session Changes in Psychological States

Table 1 shows the mean scores and their standard deviations (*SD*s) for scores from each psychological state scale at the pre- and post-session measurements. For the JBQ, mean (*SD*) total scores were 108.58 (15.51) for the wakame exercise group, 100.64 (16.66) for the push-hand sumo group, and 110.25 (17.89) for the rest group, respectively. Table 2 shows results from the two-way ANOVAs with scores from each psychological state scale as a dependent variable. Results of post hoc multiple comparisons following statistically significant interactions in these ANOVAs are further summarized in Table 3.

**Figure 3:**
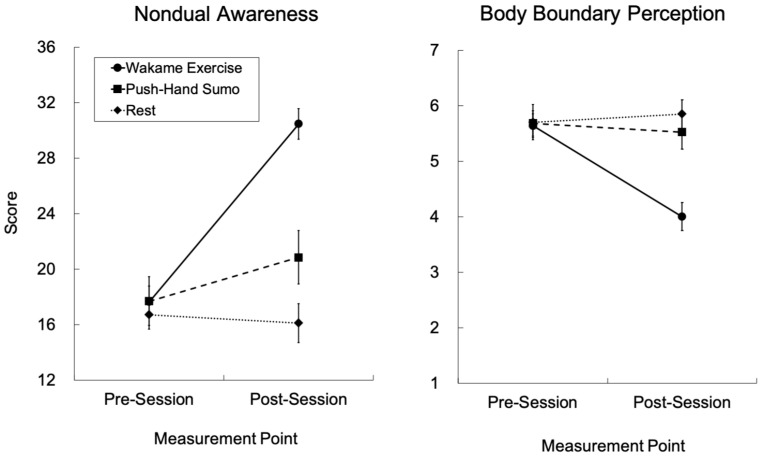
Scores from representative psychological state scales across groups and measurement points. Each graph depicts scores for the measure on nondual awareness (i.e., NADA-S) and body boundary perception (i.e., Body Boundary Perception Scale), respectively. Error bars indicate standard errors of the mean. NADA-S = Nondual Awareness Dimensional Assessment-State.

For the NADA-S, the wakame exercise group showed a statistically significant increase from pre- to post-session measurements, whereas the push-hand sumo group showed a less apparent, though statistically significant, increase. The rest group did not show significant changes through the measurement points. Regarding the Perceived Body Boundary Scale, the wakame exercise group exhibited a statistically significant decrease from pre- to post-session measurements, which shows a thinning of perceived body boundaries after the practical session. The other two groups did not show statistically significant changes in the scores through the measurement points. Scores from these two representative psychological state scales are further visualized in Figure 3. For the Creative Attitude Questionnaire, statistically significant increase from pre- to post-session measurement was observed across all groups, although the changes were numerically the smallest for the rest group. For the decentering subscale from the J-EQ, statistically significant increase from pre- to post-session measurements was observed across all groups, whereas the changes were least apparent for the rest group. Regarding the PA from the PANAS, the wakame exercise and the rest groups showed statistically significant decrease from the pre- to post-session measurements, whereas the push-hand sumo group showed a significant increase in the PA scores following practice. For the NA from the PANAS, decrease in the scores were observed across all groups after the practical session, whereas the changes were numerically the smallest for the rest group.

### 4.5. Correlations Between Psychological State Scales for the Wakame Exercise Group at the Post-Session Measurement

Table 4 shows correlation coefficients (*rs*) among the psychological state measures at the post-session measurement for the wakame exercise group. Scores from the NADA–S were significantly positively correlated those from the Creative Attitude Questionnaire and the PA from the PANAS. Scores from the Creative Attitude Questionnaire were also significantly positively correlated with those from the decentering subscale from the J-EQ. These data show significant correlations between variables on nondual awareness, creative attitude, decentering, and positive affect following practice of the wakame exercise. In addition, scores from the PA and the NA from the PANAS were significantly positively correlated with each other. No statistically significant correlations were found for the other comparisons (Table 4).

### 4.6. Correlations Between the JBQ and Psychological State Scales

Correlation coefficients (*rs*) between total scores from the JBQ and the psychological state scales for each group and measurement point are summarized in Table 1. At the pre-session measurement, scores from the JBQ were significantly positively correlated with those from the NADA-S for the wakame exercise and push-hand sumo groups. At the post-session measurement, scores from the JBQ were significantly positively correlated with those from the NADA-S for the push-hand sumo group. Post-session JBQ scores were significantly negatively correlated with those from the decentering subscale from the J-EQ for the rest group, and were significantly positively correlated with the scores from the PA for the push-hand sumo group. No statistically significant correlations were found for the other comparisons (Table 1). These data show that trait boundary perception did not significantly influence psychological states in most indices, whereas some significant correlations between trait boundary perception and psychological states were observed.

## 5. Discussion

The present study examined short-term effects of a contemplative martial arts-based paired practice, i.e., the wakame exercise, on psychological states including nondual awareness, perceived body boundaries, creative attitudes, decentering, and positive/negative affect, as well as their associations with trait boundary perception. Participants who practiced the wakame exercise showed significant increase in state nondual awareness and decrease in the salience of perceived body boundaries following practice, as well as increase in creative attitudes and decentering and decrease in both positive and negative affect. Participants from the control groups showed less apparent changes in many of these indices. Among participants who practiced the wakame exercise, state nondual awareness immediately after completing the practice was significantly positively correlated with creative attitudes, and creative attitudes were significantly positively correlated with decentering. Trait boundary perception overall did not show significant associations with psychological state measures except for a few comparisons. These results suggest that a brief paired exercise based on Japanese traditional martial arts can modulate state-level nondual awareness and self-boundary perception. Results also suggest potential relationships between nondual awareness, decentering, and creative attitudes associated with the wakame exercise.

### 5.1. Changes in Psychological States Associated with the Wakame Exercise

Results regarding psychological state changes associated with the wakame exercise overall supported our first hypothesis, except for an opposite trend observed for the positive affect. Increase in nondual awareness associated with the practical session of the wakame exercise observed in the present study is consistent with theoretical accounts of Eastern body–mind practices as methods for dissolving subject–object distinctions (Gyamtso, 1994; Nguyen, 2019). That is, the wakame exercise is deemed as a “moving meditation” based on coordinated interaction with a partner (Aoki, 1997), and this characteristic may have facilitated nondual awareness by fostering a sense of unity without strict self–other differentiation. Similar psychological effects have also been reported in mindfulness-based interventions (Hanley et al., 2020), suggesting novelty of the present findings that even a brief form of paired exercises can elicit enhanced nondual states.

Participants in the wakame exercise group also reported decreased salience of body boundary perception, indicating a reduction in the perceived separation between self and environment. Such boundary attenuation observed in the present study has been frequently linked to psychopathological states such as depersonalization disorders or complex post-traumatic stress in Western psychiatry (Nave et al., 2021). In the present study, however, the observed thinning of perceived bodily boundaries was simultaneously accompanied by increase in decentering, enhancement of creative attitudes, and decrease in negative affect, suggesting a positive link to desirable psychological statuses. These findings would be consistent with Hartmann’s (1991) notion that boundary thinness can function as an adaptive trait when core self-functions are maintained, and psychological safety is ensured.

Positive affect significantly decreased after the wakame exercise, suggesting psychological stability, whereas positive affect significantly increased following the push-hand sumo. The push-hand sumo, although involving paired physical interaction (Kurokawa, 2018), is characterized by intentional physical resistance and competitive engagement and is considered to lack contemplative dimensions. These issues may explain why the push-hand sumo introduced in the present study also enhanced creative attitudes and positive affect while having little impact on nondual awareness or perceived bodily boundaries. These findings suggest that the qualitative nature of the practices, e.g., cooperative versus competitive engagement with others, may have differentially influenced psychological outcomes. That is, the results may highlight the distinctive nature of the wakame exercise as a traditional martial art-based contemplative practice.

### 5.2. Nondual Awareness, Creative Attitudes, and Decentering Associated with the Wakane Exercise

Among the wakame exercise group, statistically significant positive correlations were found at the post-session measurement between nondual awareness and creative attitudes, creative attitudes and decentering, and nondual awareness and positive affect. These correlations may potentially be consistent with the psychoanalytic concept of “creative regression” (Kris, 1952; Schafer, 1954), which assumes that a temporary loosening of ego boundaries can facilitate novel associations and creative insights. In contrast, no statistically significant correlations were observed between creative attitudes and positive affect. This suggests that enhanced creative attitudes after the wakame exercise may not be accompanied by increased positive affectivity. Thus, our second hypothesis was partially supported, although zero-order correlations are not sufficient to determine causal relationships between these psychological constructs.

### 5.3. Trait Boundary Perception and Psychological States

Regarding trait boundary thinness, as measured by the JBQ, statistically significant correlations with psychological state measures were not observed in most comparisons. Among the wakame exercise group, a statistically significant correlation between the JBQ and state nondual awareness was found before starting the practice, although the correlations were not significant at the post-session measurement. These results indicate that nondual awareness after the practice of the wakame exercise was not preferentially experienced by individuals with thinner trait boundaries; rather, the wakame exercise facilitated nondual awareness regardless of baseline trait boundary tendencies. Therefore, our third hypothesis was not supported by the results.

### 5.4. Comparison with Previous Studies and Novel Perspectives

Findings obtained from the present study are consistent with previous studies reporting mental health benefits of martial arts and contemplative practices (Ciaccioni et al., 2023; Priddy et al., 2018; Wang et al., 2013). Our study extends these literature by demonstrating that even a short-term, paired martial arts intervention can elicit measurable state-level changes in nondual awareness and self-boundary perception, emphasizing the role of cooperative interaction rather than physical activity alone. Importantly, the wakame exercise employed in the present study is a contemplative bodywork based on Japanese traditional martial arts, characterized by slow movements performed in pairs and involving gentle, reciprocal physical contact. These features of the wakame exercise distinguish it from solitary physical exercises or purely athletic forms of martial arts. The changes in psychological states observed in the presnet study may thus reflect a multifaceted nature of such embodied practices, which integrate physical, psychological, psychosocial, and spiritual components within a holistic framework of health. These notions would also be parallel to what Vergeer et al. (2021) describe as holistic movement practices (HMPs). The finding that such multidimensional effects were observed even in a brief, single-session intervention among beginners should represent a novel contribution to the existing body of research in this frontier.

### 5.5. Limitations and Future Perspectives

Despite the novel findings and perspectives outlined above, the present study has several apparent limitations. First, the intervention consisted of a single 40 min session, which limits conclusions about long-term effects. Future studies should employ repeated or longitudinal interventions to examine whether changes in nondual awareness, perceived body boundaries, and creative attitudes are sustained over time. Second, a priori power analysis to estimate the optimal sample size was not conducted prior to data collection, and post hoc estimation indicated that the achieved sample size was underpowered for some outcomes. As Cohen (1988) noted, post hoc power calculations have limited interpretive value and should be considered with caution. Nevertheless, it would be reasonable to note that future work may include larger, fully powered samples to provide more robust statistical evidence. Third, researchers who analyzed the data following data collection were not blinded to group assignment when conducting the study, which may have introduced potential bias. This limitation was inherent to the fact that data collection was conducted as part of the university classes; however, future studies may consider employing better procedures for blinding. Fourth, all outcomes relied on self-reports, and thus causal interpretations should be made with caution due to the single-session design and correlational analyses. Future research may employ longitudinal designs and incorporate physiological indices (e.g., heart rate variability) to better assess causal mechanisms. Fifth, the NADA-S (Hanley et al., 2018) used to assess state nondual awareness lacked an official Japanese version, and was thus administered in a provisional translation. The Creative Attitudes Questionnaire was also researcher-developed based on the literature (Onda, 1995) and is not fully validated. It is desirable to further develop and standardize culturally appropriate measures for these psychological constructs. Sixth, although the push-hand sumo was introduced as an exercise theoretically comparable to the wakame exercise in terms of exercise intensity, no objective indicators (e.g., physiological or kinematic measures) were obtained to confirm that exercise intensity for these activities were actually equivalent. It is thus a challenge to include both psychological and physiological measures to objectively assess intensity of these exercises experienced by the participants. Finally, the sample included only Japanese university students, which should restrict generalizability of the findings. Future research may promisingly involve samples from more diverse cultural and age backgrounds to uncover whether the observed effects may generalize beyond the present population.

### 5.6. Practical Implications

Despite their exploratory nature, results from the present study suggests that a brief paired contemplative practice based on Japanese martial arts may potentially be beneficial for enhancing state-level nondual awareness and creativity in educational settings. The Japanese cultural context, including culturally embedded notions of harmonious, relational self, likely influenced the feasibility, safety, and outcomes of the intervention. Therefore, participants from cultures with different conceptions of self (e.g., more individualistic values) may experience different effects as a result of the comparable practice. Although the present study focused on the wakame exercise, other Eastern mind–body practices (e.g., meditation, qigong, yoga, Tai Chi) may either similarly or differentially impact nondual awareness, self–other boundary perception, and related psychological outcomes. Comparative studies in this frontier may explore how multiple practices based on Eastern traditions influence boundary perception and psychological states.

## 6. Conclusions

To conclude, the present study provides initial evidence that a single-session practice of the wakame exercise, a contemplative paired bodywork based on Japanese traditional martial arts, can induce altered psychological states including enhanced nondual awareness, reduced salience of perceived self–other boundaries, and increased creative attitudes. Among individuals who completed the wakame exercise, state nondual awareness was positively associated with creative attitudes, and creative attitudes were positively associated with decentering, suggesting that temporary boundary transformation may underlie the psychological benefits of such practices. These results were found regardless of baseline boundary perception at the trait level, suggesting that the observed changes primarily reflect the effects of engaging in the exercise itself, rather than pre-existing differences in boundary thinness. Future research may involve both longitudinal studies to examine the effects of continued practice and cross-cultural comparisons to examine generalizability of the findings.

## Figures and Tables

**Table 1:** Mean (*SD*) scores from the psychological state scales and their correlations with the JBQ scores shown for each group and at the pre- and post-session measurements, respectively.

Psychological Scale	Group	Score		Correlation with the JBQ (*r*)	
		Pre-Session	Post-Session	Pre-Session	Post-Session
NADA-S	Wakame exercise	17.64 (5.42)	30.46 (5.27)	0.491 *	0.118
	Push-hand sumo	17.68 (9.02)	20.84 (9.83)	0.530 **	0.418 *
	Rest	16.70 (4.68)	16.10 (6.44)	0.122	0.038
Body Boundary Perception	Wakame exercise	5.64 (0.22)	4.00 (0.25)	0.103	–0.101
	Push-hand sumo	5.68 (1.18)	5.52 (1.56)	−0.238	−0.153
	Rest	5.70 (1.46)	5.85 (1.18)	−0.056	−0.058
Creative Attitude	Wakame exercise	27.00 (5.54)	29.56 (4.70)	0.272	0.267
	Push-hand sumo	26.12 (4.89)	28.36 (6.24)	0.157	−0.047
	Rest	27.15 (3.98)	28.55 (4.25)	0.220	0.127
Decentering	Wakame exercise	29.88 (6.39)	38.68 (6.88)	0.099	–0.060
	Push-hand sumo	28.84 (6.47)	39.08 (8.31)	0.226	0.135
	Rest	31.05 (6.93)	36.30 (7.45)	−0.233	−0.466 *
PANAS: PA	Wakame exercise	20.17 (6.57)	16.96 (5.56)	0.155	0.073
	Push-hand sumo	21.48 (7.58)	26.88 (8.47)	−0.054	0.426 *
	Rest	19.90 (7.29)	16.65 (7.85)	−0.219	−0.158
PANAS: NA	Wakame exercise	16.58 (6.49)	10.00 (3.49)	−0.016	0.254
	Push-hand sumo	19.00 (7.50)	11.72 (4.17)	0.265	0.093
	Rest	14.50 (6.76)	10.90 (4.45)	−0.165	−0.093

* *p* < 0.05; ** *p* < 0.01.

**Table 2:** Results of the two-way ANOVAs for each psychological state scale.

Psychological Scale	Main Effect: Group			Main Effect: Measurement Point			Interaction: Group × Measurement Point		
	*F*_2, 67_	*p*	Partial *η*^2^	*F*_1, 67_	*p*	Partial *η*^2^	*F*_2, 67_	*p*	Partial *η*^2^
NADA-S	7.468	0.001 **	0.19	44.633	<0.001 ***	0.41	26.251	<0.001 ***	0.45
Body Boundary Perception	4.420	0.016 *	0.12	13.203	<0.001 ***	0.17	13.053	<0.001 ***	0.29
Creative Attitude	0.313	0.732	0.01	19.664	<0.001 ***	0.23	0.518	0.598	0.02
Decentering	0.046	0.955	0.00	189.775	<0.001 ***	0.74	5.988	0.004 **	0.15
PANAS: PA	5.847	0.005 **	0.15	0.217	0.643	0.00	17.659	<0.001 ***	0.35
PANAS: NA	1.744	0.183	0.05	85.145	<0.001 ***	0.56	2.999	0.057	0.08

* *p* < 0.05; ** *p* < 0.01; *** *p* < 0.001.

**Table 3:** Results of post hoc comparisons between the pre- and post-session measurements following the two-way ANOVAs.

Psychological Scale	Group	*t* (*p*)	*d*
NADA-S	Wakame exercise	−9.613 (<0.001) ***	−1.74
	Push-hand sumo	−2.526 (0.014) *	−0.55
	Rest	0.429 (*n.s.*)	0.082
Body Boundary Perception	Wakame exercise	6.234 (<0.001) ***	1.25
	Push-hand sumo	0.650 (*n.s.*)	0.17
	Rest	−0.545 (*n.s.*)	−0.12
Decentering	Wakame exercise	−8.997 (<0.001) ***	−1.22
	Push-hand sumo	−10.470 (<0.001) ***	−1.92
	Rest	−4.801 (<0.001) ***	−0.73
PANAS: PA	Wakame exercise	2.655 (0.010) **	0.42
	Push-hand sumo	−4.683 (<0.001) ***	−0.99
	Rest	2.521 (0.014) *	0.44

* *p* < 0.05; ** *p* < 0.01; *** *p* < 0.001. *n.s.* = non-significant.

**Table 4:** Correlation coefficients (*rs*) between psychological state scales at the post-session measurement for the wakame exercise group.

Psychological Scale	1	2	3	4	5
1. NADA-S	–				
2. Body Boundary Perception	−0.200	–			
3. Creative Attitude	0.533 *	−0.017	–		
4. Decentering	0.394	−0.065	0.538 **		
5. PANAS: PA	0.492 *	0.176	0.132	0.230	–
6. PANAS: NA	0.184	−0.162	−0.056	0.096	0.582 **

* *p* < 0.05; ** *p* < 0.01.

## Data Availability

The data that support the findings of this study are available from the corresponding author upon reasonable request.
